# Supplementary material for: Experimental identification of topological defects in 2D colloidal glass
Source: Nat Commun. 2025 Jan 2;16:55. doi: 10.1038/s41467-024-54857-z (PMC11697214; doi:10.1038/s41467-024-54857-z)
Supplement: Supplementary file 1 — Supplementary Information [file 41467_2024_54857_MOESM1_ESM.pdf]

Supplementary Information

In this Supplementary Information (SI) we provide additional details about the experimental conditions and setup, a brief description of the structure and dynamics of the system, and some simulation results to complement the experimental observation.

Additional information related to the experiment

Experimental information has been described in detail in [1], but we briefly outline here the crucial steps. Our system consists of a binary colloidal mixture having paramagnetic properties, suspended at a flat air-water interface due to gravity. The tilt and interface position is well controlled throughout the experiment to realize a perfect two-dimensional system. In the presence of an external magnetic field, for the typical inter-particle separation at room temperature, the dominating interaction between particles is dipole-dipole interaction.

Superparamagnetic colloidal particles: The colloidal particles are polystyrene beads doped with magnetite (Fe_2O_3) and sealed with a thin layer of epoxy. In the absence of an external magnetic field, net magnetization in each colloidal particle is zero. There are two types of particles (diameters: $d_A = 4.5 \pm 0.05\ \mu\text{m}, d_B = 2.8\ \mu\text{m}$; mass density: $\rho_A = 1.5\ \text{g/cm}^3, \rho_B = 1.3\ \text{g/cm}^3$ and magnetic susceptibility per particle: $\chi_A = 6.22 \times 10^{-11}\ \text{Am}^2/\text{T}, \chi_B = 6.6 \times 10^{-12}\ \text{Am}^2/\text{T}$). Particles observed under transmission electron microscopy show that the magnetite is uniformly distributed over each particle volume and bigger particles are quite monodisperse. Smaller particles may have small polydispersity.

Suspensions of bigger and smaller particles are prepared separately and after treating properly to make sure that the suspension is uniform and there is no particle aggregation, they are mixed properly to get the mixture with the desired composition $\xi = N_A/(N_A + N_B)$; N_A and N_B are the numbers of bigger and smaller species respectively. For the present colloidal system, we have an equal number of bigger and smaller species, i.e., $\xi \approx 0.5$.

Formation of air-water interface and its control: A cylindrical hole of diameter 6 mm and depth 1 mm is created in a glass plate. A water droplet hangs inside this cylindrical geometry due to surface tension. The bottom air-water interface hosts the suspension of the binary colloidal mixture after sedimentation due to gravity, while the top of the sample cell is properly sealed. A computer-controlled microsyringe is attached which controls the volume of the pended drop to ensure a flat interface. A schematic diagram of this setup is shown in Fig. 1 of the main text. The colloidal particles can freely diffuse over the interface. In other experimental setups where such a system is realized over a substrate, it is difficult to avoid the pinning of a few particles. The curvature and inclination of the interface have been adjusted over months to achieve a gradient-free density profile. The inclination is controlled with an external sensor down to $\pm 10^{-6}$ rad. There are copper coils that generate an almost uniform magnetic field in the plane of the sample (deviation is less than 0.5% in the field of interest), including a compensation of earth's magnetic field.

Video microscopy is employed to visualize the particles from the below side of the sample and an 8-bit charge-coupled device camera records the gray scale images of particles which are analyzed by a computer. There are approximately 10^5 particles in the sample but the camera has the field of view of $\approx 1\text{mm}^2$ $(1158 \times 865 \mu\text{m}^2)$ which can capture about 2300 particles. In this setup, the particle trajectories can be recorded for several days with the spatial resolution of ≈ 50 nm and a time resolution of ≈ 1 s.

For the analysis presented in the main text, we have utilized 1000 configurations sampled over a period of 2772 seconds. Throughout the experiments, room temperature is maintained.

Structure and dynamics of the colloidal system

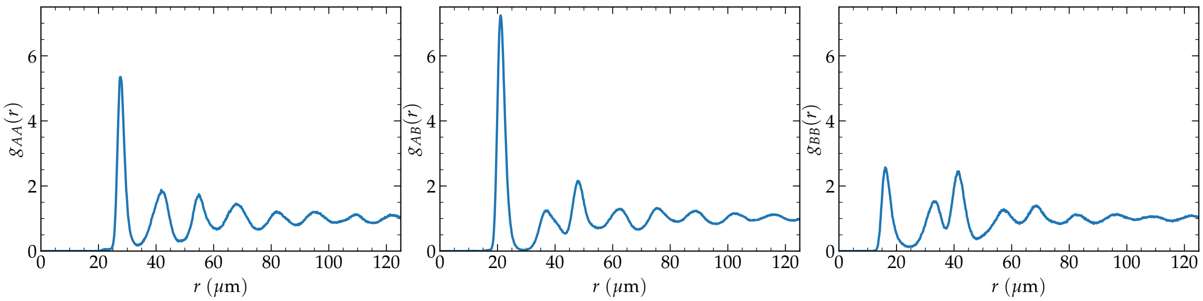

Supplementary Figure 1: Structure of the system. Partial pair correlation functions $g_{AA}(r), g_{AB}(r)$ and $g_{BB}(r)$ calculated for the samples used in the study.

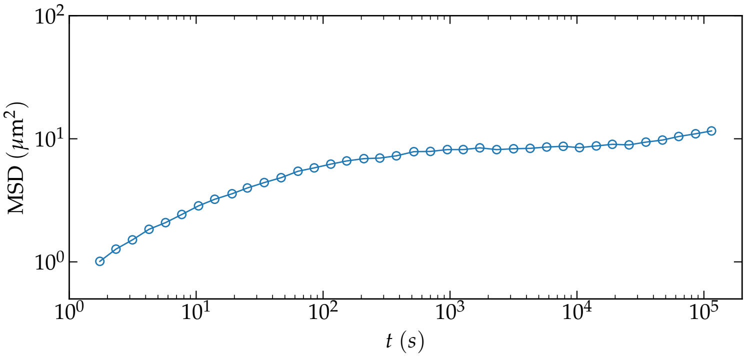

Supplementary Figure 2: Dynamics of the system. Mean squared displacement (MSD) as a function of time.

As discussed in the main article, Γ, which is the ratio of magnetic dipole-dipole interaction energy to thermal energy (k_BT):

$$\Gamma = \frac{\mu_0}{4\pi}\frac{(\pi n)^{3/2}}{k_BT}[\xi\chi_B + (1-\xi)\chi_A]^2. \quad (1)$$

Here, n is the area density computed via Voronoi tesselation. Γ for the sample used in this study is 423. At this value of Γ, the structure and dynamics of the system are of a typical glass. We discuss in the next paragraph about the pair-correlation function and mean-squared displacement (MSD) of the sample, to quantify structure and dynamics.

Partial pair-correlations in Supplementary Fig. 1 indicate that the mixture is homogeneous and the structure is disordered. MSD plotted in Supplementary Fig. 2 has an extended plateau, this means that the dynamics of the system is largely arrested. These features establish the typical glassy behavior of the samples used in this study.

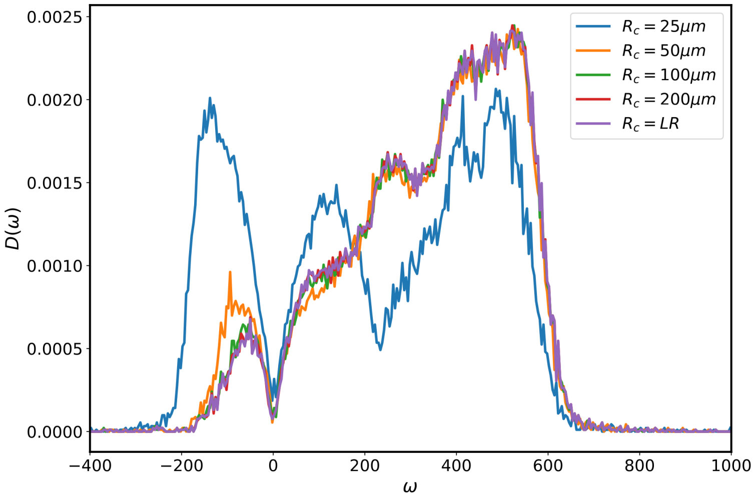

Supplementary Figure 3: vDOS for various cut-off ranges. $D(\omega)$ has been plotted against ω for different cut-off interaction range R_c.

Robustness of vibrational density of states

The dipole-dipole interaction potential in this experimental system is long-ranged (LR). To ensure the robustness of our findings, we tested our method by varying the cut-off distances, R_c, for the interaction potential. In supplementary Fig. 3, we present $D(\omega)$ for different values of R_c. The rapid convergence of $D(\omega)$ towards the long-range (LR) case as R_c increases clearly demonstrates the robustness of our results.

To evaluate also the impact of polydispersity, we considered two different scenarios. First, we introduced a 10% random polydispersity in the particle masses. Second, we imposed a 10% random error in the inter-particle distances, leading to a rougher potential surface. We then computed the vibrational density of states for both scenarios and compared them with our

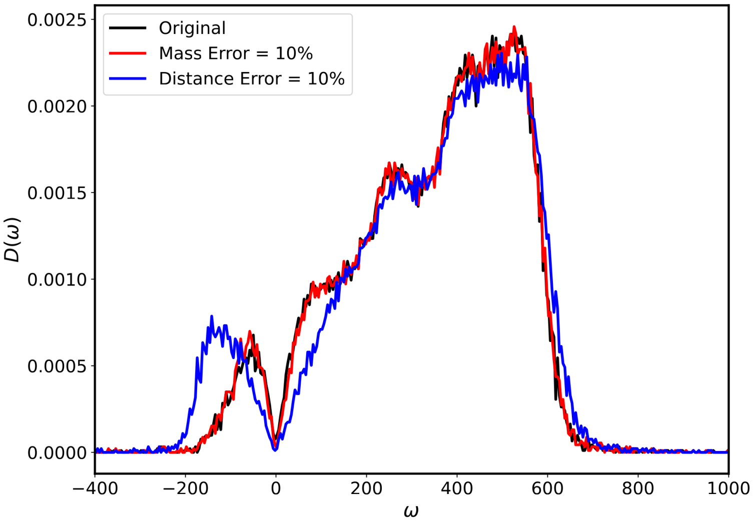

Supplementary Figure 4: vDOS for polydispersity in masses and errors in distance measurements The vibrational density of states $D(\omega)$ versus ω for the original long-range (LR) interaction, including scenarios with a 10% random polydispersity in particle masses and a 10% random error in distance measurements.

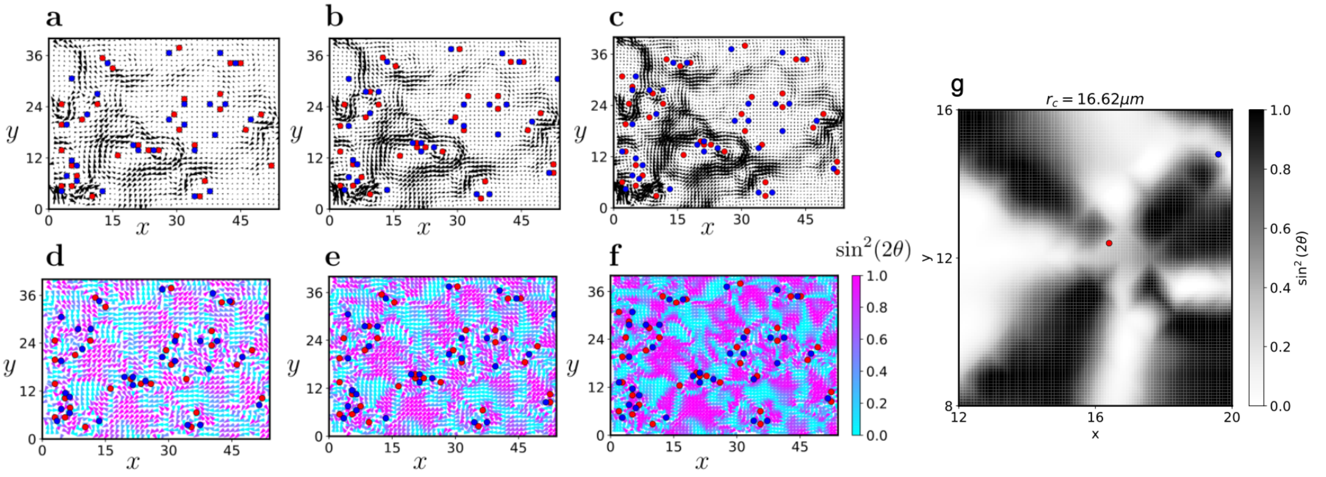

Supplementary Figure 5: Eigenvector field with defects for different grid lengths. a-c, Interpolated eigenvector field for grid lengths of 24.94 μm with rectangular lattice (45 × 33), 20.78 μm (54 × 40), and 16.62 μm (67 × 50), respectively. **d-f,** Corresponding Schlieren textures for panels **a-c**. The color intensity is mapped based on $\sin^2(2\theta)$ (θ being phase of eigenvector field), analogous to Schlieren patterns. **g**, A portion of the system for $r_c = 16.62$ μm is shown in the standard Schlieren textures using a diffrent color intensity. Defects with winding numbers +1 and -1 are marked by red and blue circles, respectively. For clearer visualization, the eigenvector field is multiplied by 30 in panels **a-c**, and normalized to a magnitude of 0.9 in panels **d-f**. In all cases, the field of view is rectangular ($54a \times 40a$, where $a = 20.78$ μm). These data are for eigenfrequency $\omega = 1.53$ Hz.

long-range (LR) data. The comparison is presented in supplementary Fig. 4. Our results show that mass polydispersity has a very minimal effect on the vibrational properties. While the random error in distance measurements has a somewhat stronger impact, the nature of $D(\omega)$ versus ω changes only slightly, i.e., an increase in imaginary frequency modes. Based on this new analysis and data, we believe that the vibrational characteristics are quite robust, even in the presence of small experimental measurement errors.

Robustness of topological defects

We conducted defect analysis by varying the grid size by 20%. The data presented in the main text correspond to a grid size of 20.78 μm, with a rectangular mesh of (54×40). To test the robustness of the numerical methods, we also interpolated the

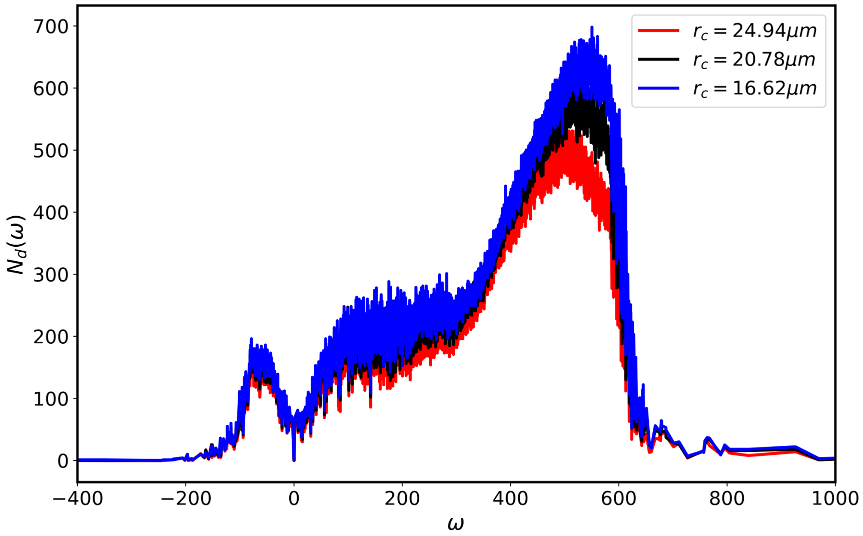

Supplementary Figure 6: Behavior of defect numbers for different grid lengths. Total number of defects (N_d) versus ω for various interpolation grid lengths.

eigenvector field on rectangular grids with dimensions of 45×33 (grid length 24.94 μm) and 67×50 (grid length 16.62 μm). In Supplementary Fig. 5, we display the eigenvector field for a low frequency of $\omega = 1.53$ Hz. Across all three grid lengths, we observe consistent defect locations (Supplementary Fig. 5a–c). For enhanced visualization, the same configurations are plotted in Schlieren patterns [2] in Supplementary Fig. 5d–f, where the color bar represents $\sin^2(2\theta)$, with θ being the phase of the eigenvector field. In these Schlieren textures, defect locations are easily identified by the merging of different colors, as shown in Supplementary Fig. 5d–f.

In Supplementary Fig. 6a, we present the variation in the total number of defects across different grid lengths. This consistent defect behavior across varying interpolation grid lengths supports the robustness of the results. The observed differences in N_d across specific frequency regions for varying r_c can be simply understood as follows. The loop integral of the local phase $q = 1/(2\pi)\oint d\theta$ counts by definition the total topological charge inside such loop. When the grid size becomes larger, and the analysis more coarse grained, the probability of having several microscopic topological defects inside the loops increases. As a consequence, it is more likely to have a situation with two (or more) defects with opposite charges inside the loop. This situation would output $q = 0$ and therefore "no defects" inside that region would be detected. This is why by making the grid larger the "number of defects" decreases. Notice also how this difference is more marked at large frequencies where the number of defects becomes very large and their spatial distance smaller, increasing the probability of having cases as the one just described. In other words, counting the topological charge inside loops coincides with counting the number of topological defects only when the smallest grid size is chosen. Despite this important observation, for small variations of the grid size the changes are minimal. This consistent defect behavior across different (nearby) r_c values demonstrates the robustness of the results presented in the manuscript.

Softness field

To identify the optimal cutoff value in the determination of softness field, we calculate the softness parameter per particle with different choices of N_m corresponding to different ω_{cut}, *i.e.*, all the real vibrational modes below ω_{cut} (supplementary Fig. 7). We observe that a small cutoff, $\omega_{cut} = 25$, produces significantly different results when compared the case of $\omega_{cut} = 50$ used in the main text. Nevertheless, if we increase the cutoff further then the softness parameter do not present significant changes and the numerical results converge. Hence, $\omega_{cut} = 50$ is the optimal cutoff value for this numerical procedure. For the calculations in the main text, N_m is considered such that $\omega_{\text{cut}} = 50$.

2D Lennard-Jones glass model

We have also performed a set of simulations of a two-dimensional Lennard-Jones glass as that studied by Wu et al. in [3]. We have extended their analysis of vibrational spectra and topological features to finite temperature systems, to better compare with our experimental setup. We aim to understand whether the low-frequency behavior of N_d, *i.e.*, the total number of topological defects changes for finite temperature glasses. We perform additional simulations of thermal glass with the athermal one using

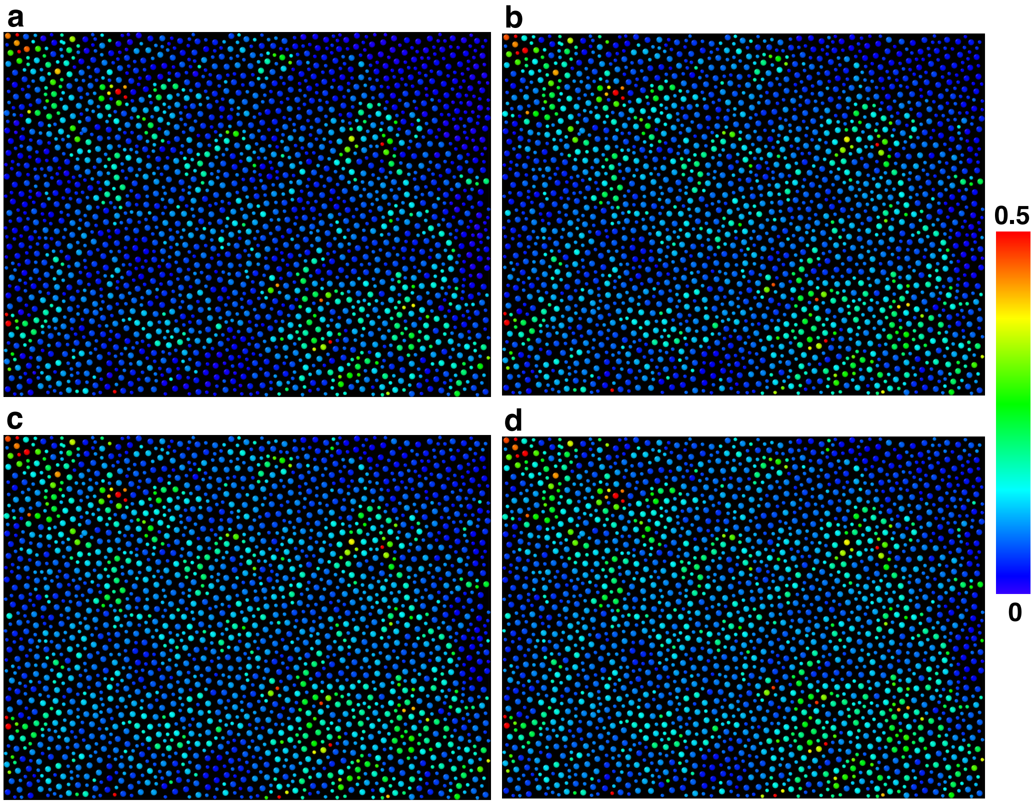

Supplementary Figure 7: Softness field. a-d, Snapshot of the epxerimental system with particles colored as per their respective value of softness for $\omega_{\mathrm{cut}} = 25, 50, 75, 100$.

LAMMPS [4]. Thermalized glassy configurations are prepared by cooling the high-temperature glass at $T_i = 5.0$ to the target temperatures $T = 0.1, 0.2, 0.4$ over 10^7 time step ($\delta t = 0.001$) and then annealed for further 10^6 number of time steps. For the preparation of athermal configurations, we have followed the same protocol as discussed in [3]; energy of $T = 0.1$ glassy configuration is minimized using conjugate gradient algorithm.

References

1. Ebert, F., Dillmann, P., Maret, G. & Keim, P. The experimental realization of a two-dimensional colloidal model system. Rev. Sci. Instruments **80** (2009).
2. Hoffmann, K. B. & Sbalzarini, I. F. Robustness of topological defects in discrete domains. Phys. Rev. E **103**, 012602, DOI: 10.1103/PhysRevE.103.012602 (2021).
3. Wu, Z. W., Chen, Y., Wang, W.-H., Kob, W. & Xu, L. Topology of vibrational modes predicts plastic events in glasses. Nat. Commun. **14**, 2955, DOI: 10.1038/s41467-023-38547-w (2023).
4. Plimpton, S. Fast parallel algorithms for short-range molecular dynamics. J. Comp. Phys **117**, 1–19 (1995). See also: http://lammps.sandia.gov.
